# Patterns and Predictors of Multimorbidity in the Azar Cohort

**DOI:** 10.34172/aim.2023.02

**Published:** 2023-01-01

**Authors:** Mohammdhossein Somi, Alireza Ostadrahimi, Neda Gilani, Arash Haji Kamanaj, Sina Hassannezhad, Elnaz Faramarzi

**Affiliations:** ^1^Liver and Gastrointestinal Diseases Research Center, Tabriz University of Medical Sciences, Tabriz, Iran; ^2^Nutrition Research Center, Tabriz University of Medical Sciences, Tabriz, Iran; ^3^Department of Statistics and Epidemiology, Faculty of Health, Tabriz University of Medical Sciences, Tabriz, Iran; ^4^Student Research Committee, Tabriz University of Medical Sciences, Tabriz, Iran

**Keywords:** Chronic disease, Cohort study, Multimorbidity, Persian cohort, Sleep habits

## Abstract

**Background:**

The co-existence of chronic diseases (CDs), a condition defined as multimorbidity (MM), is becoming a major public health issue. Therefore, we aimed to determine the patterns and predictors of MM in the Azar Cohort.

**Methods:**

We evaluated the prevalence of MM in 15,006 (35–70-year old) subjects of the Azar Cohort Study. MM was defined as the co-existence of two or more CDs. Data on the subjects’ socioeconomic status, demographics, sleeping habits, and physical activity were collected using questionnaires.

**Results:**

The overall prevalence of MM was 28.1%. The most prevalent CDs, in decreasing order, were obesity, hypertension, depression, and diabetes. Obesity, depression, and diabetes were the most co-occurring CDs. The MM risk increased significantly with age, illiteracy, and in females. Also, the subjects within the lowest tertile of physical activity level (OR=1.89; 95% CI: 1.75–2.05) showed higher MM risk than those with the highest level of physical activity. Findings regarding current smoking status indicated that being an ex-smoker or smoker of other types of tobacco significantly increased the risk of MM.

**Conclusion:**

The reduction of MM is possible by promoting public health from an early age among people of various socioeconomic conditions. It is vital to offer the necessary health support to the aging population of Iran.

## Introduction

 Multimorbidity (MM) is often described as the co-existence of two or more chronic diseases (CDs),^1^ representing a global problem increasing in prevalence.^2^ The prevalence of MM varies from 12.9% in the general population to 95.1% in older people.^3^ Individuals with MM often have adverse health outcomes, including disability,^4^ decreased quality of life,^5^ and functional decline.^6^ Moreover, it increases the chance of premature death.^7^ As a result, MM significantly affects healthcare expenditures, resource allocation, and disease management.^8-10^ Research on MM is essential yet challenging due to lack of agreement on its definition.^11^

 Many risk factors (age, obesity, smoking, hypertension, etc) are linked with MM.^3^ Different levels of exposure to these risk factors may explain why MM and its patterns vary among demographic subgroups.^12^ Moreover, many cohort MM studies have focused on individuals aged above 65. Therefore, it is impossible to determine the patterns of MM in primary ages. Understanding the general framework of MM in accordance with well-known risk factors can assist in finding direct and indirect relationships between them. This will enable predicting their occurrence sequence in people with different characteristics. Moreover, using this information, we will be able to identify and prevent the vicious cycle of the diseases at an appropriate and reversible point.^13^

 In light of the mentioned factors, this study sought to determine the prevalence of MM according to different demographic factors in the Azar Cohort Study population. Thereafter, this study aimed to express a comprehensive relationship pattern between various diseases (such as coronary heart diseases [CHD], cancers, asthma, and other frequent diseases) and their risk factors.

## Materials and Methods

 This cross-sectional study evaluated the prevalence of MM in 15 006 subjects of the Azar Cohort Study. In that study, the list and medical records of all subjects aged 35–70 years were provided by the Shabestar Health Center (East Azerbaijan Province, Iran). Those included resided in the Shabestar County for at least nine months. According to the study protocol, almost all eligible subjects in the small cities and villages were invited. In larger cities, more than 60% of the target population were invited. Individuals with severe psychiatric or physical illness were excluded from the study. Finally, 15,006 subjects were recruited in the Azar Cohort Study. All subjects provided written informed consent. The study is part of the Large Prospective Epidemiological Research Studies in Iran (PERSIAN) cohort.^14^ The pilot and the enrollment phases for this study were launched in 2014, and the study concluded in 2017. Comprehensive details about the Azar Cohort Study are provided in another published article.^15^

###  Multimorbidity Definition 

 MM was defined as the co-existence of two or more CDs, including hypertension, diabetes, CHD, stroke, chronic obstructive pulmonary diseases (COPD), cancers (gastrointestinal, breast, prostate, skin, bladder, lung, head and neck, or hematopoietic), depression, fatty liver, rheumatoid disease, and obesity. In the questionnaires, participants were considered to have these diseases when they answered yes to the following question: “Has any doctor ever told you that you have …?” Moreover, obesity was defined by a body mass index (BMI) ≥ 30 kg/m^2^.

###  Anthropometric Measurements

 The weight and height of all subjects were measured, and BMI was determined using the standard formula: weight (kg)/height (m^2^). The anthropometric measurements are described in detail elsewhere.^14^

###  Demographic Information 

 Information regarding age, gender, education level, marital status, smoking status, and sleeping habits were collected using questionnaires. People who smoked at least one cigarette per day for more than six months continuously were defined as smokers, those who had ceased smoking at least a year ago were regarded as ex-smokers, subjects who had never smoked were labeled as non-smokers, and people who smoked hookah, water pipe, pipe and chewed nass were categorized as users of other tobacco products.

 Socioeconomic status was evaluated using Wealth Score Index (WSI), calculated by multiple correspondence analysis. Ownership of a variety of durable assets (e.g., dishwasher, car, and television), household conditions (e.g., number of rooms, type of ownership), and education level were used in the calculation of the WSI for each participant. Participants of the study were categorized into five WSI quintiles, from the lowest (1st quintile) to the highest (5th quintile). In this study, the participants’ daily activity was evaluated using a questionnaire. For this purpose, a criterion called the metabolic equivalent (MET) was employed. Each MET is equal to the amount of energy that a person consumes relative to their weight. For instance, one MET is equal to the amount of oxygen used by a person while resting per kilogram of their body weight per minute, which is 3.5 mL of oxygen, and 4 METs equal 14 mL of oxygen used per kilogram of their body weight per minute. Through this criterion, we obtained the level of activity for each person.

###  Statistical Analysis

 The normality of data was assessed using the Kolmogorov-Smirnov test and descriptive statistics. The mean (standard deviation) was reported for quantitative data, while the frequency (percentage) was reported for qualitative data. Quantitative variables included age and BMI, while qualitative variables included gender, marital status, education level, residential region, smoking status, WSI, and sleep duration. The one-way ANOVA, chi-square, and Kruskal-Wallis H tests were used to compare quantitative, qualitative, and categorical qualitative variables between MM classifications, respectively. MMs were classified into four groups: 0 (no CDs), 1 (one CD), 2 (two CDs), 3 (3 CDs), and 4 ( ≥ 4 CDs). Moreover, WSI, METs, and sleep duration were categorized into quintiles, tertiles, and quartiles, respectively. To determine the predictors of MM, an ordinal logistic regression analysis was conducted (Model 1: unadjusted, Model 2: adjusted for gender, age, marital status, education level, and socioeconomic status if applicable). Odds ratios (ORs) and the 95% confidence intervals (CIs) were estimated. Statistical significance was considered when *P* < 0.05. Data analysis was conducted using SPSS (SPSS Inc., Chicago, IL, version 20).

## Results

 Our findings indicated that in the cohort (n = 15 006), 32.8% had at least one CD (Table 1). MM (CD ≥ 2) was seen in 28.1% of the study population. The most prevalent CDs were obesity (37.6%), hypertension (20.2%), depression (17.1%), and diabetes (11.6%). In addition, obesity, depression, and diabetes were the most co-occurring CDs in our population (Table 2). As indicated in Table 3, hypertension, obesity, depression and diabetes were the most prevalent chronic diseases in subjects with 2 or more disaeses.

**Table 1:** Prevalence of Chronic Diseases and Multimorbidity Classification in the Azar Cohort Population

**Variable **	**Number**	**Percent**
Hypertension	3039	20.2
Diabetes	1746	11.6
CHD	738	4.9
Obesity	5641	37.6
Stroke	119	0.8
Fatty liver	734	4.9
Rheumatoid	514	3.4
Cancers	85	0.6
Depression	2562	17.1
COPD	538	3.6
Number of chronic diseases multimorbidity classification		
0	5865	39.1
1	4923	32.8
2	2547	17
3	1151	7.7
≥ 4	520	3.4

CHD, Coronary heart diseases; COPD, Chronic obstructive pulmonary diseases.

**Table 2:** The Most Common Co-occurring Chronic Diseases in the Azar Cohort Population

	**Hypertension**	**Diabetes**	**CHD**	**Obesity**	**Stroke**	**Fatty liver**	**Rheumatoid**	**Cancers**	**Depression**
	**No. (%)**	**No. (%)**	**No. (%)**	**No. (%)**	**No. (%)**	**No. (%)**	**No. (%)**	**No. (%)**	**No. (%)**
Diabetes	823 (27.1)	-	221 (29.9)	868 (15.4)	25 (21)	188 (25.6)	75 (14.6)	12 (14.1)	381 (14.9)
CHD	460 (15.1)	221 (12.7)	-	329 (5.8)	27 (22.7)	54 (7.4)	46 (8.9)	3 (3.5)	165 (6.4)
Obesity	1582 (52.1)	868 (49.8)	329 (44.6)	-	48 (40.3)	44 (60.1)	236 (46)	34 (40)	1144 (44.7)
Stroke	78 (2.6)	25 (1.4)	27 (3.7)	48 (0.9)	-	7 (1)	6 (1.2)	0 (0)	43 (1.7)
Fatty liver	259 (8.5)	188 (10.8)	54 (7.3)	441 (7.8)	7 (5.9)	-	45 (8.8)	10 (11.8)	220 (8.6)
Rheumatoid	134 (4.4)	75 (4.3)	46 (6.2)	236 (4.2)	6 (5)	45 (6.1)	-	6 (7.1)	149 (5.8)
Cancers	20 (0.7)	12 (0.7)	3 (0.4)	34 (0.6)	0 (0)	10 (1.4)	6 (1.2)	-	20 (0.8)
Depression	861 (28.3)	381 (21.8)	165 (22.4)	1144 (20.3)	43 (36.1)	220 (30)	149 (29)	20 (23.5)	-
COPD	185 (6.1)	89 (5.1)	51 (6.9)	241 (4.3)	10 (8.4)	38 (5.2)	39 (7.6)	4 (4.7)	130 (5.1)

CHD, Coronary heart diseases; COPD, Chronic obstructive pulmonary diseases.

**Table 3:** Prevalence of Chronic Diseases Stratified by Multimorbidity

	**0 (n=5865)**	**1 (n=4923)**	**2 (n=2547)**	**3 (n=1151)**	**≥4 (n=520)**
	**No. (%)**	**No. (%)**	**No. (%)**	**No. (%)**	**No. (%)**
Hypertension	0 (0)	569 (11.6)	1157 (45.4)	847 (73.6)	466 (89.61)
Diabetes	0 (0)	375 (7.6)	536 (21)	491 (42.7)	344 (66.15)
CHD	0 (0)	106 (2.2)	201 (7.9)	228 (19.8)	203 (39.03)
Obesity	0 (0)	2648 (53.8)	1673 (65.7)	863 (75)	457 (87.88)
Stroke	0 (0)	17 (0.3)	34 (1.3)	26 (2.3)	42 (8.07)
Fatty liver	0 (0)	116 (2.4)	238 (9.3)	203 (17.6)	177 (34.03)
Rheumatoid	0 (0)	136 (2.8)	163 (6.4)	120 (10.4)	95 (18.26)
Cancers	0 (0)	24 (0.5)	27 (1.1)	23 (2)	11 (2.11)
Depression	0 (0)	788 (16)	906 (35.6)	530 (46)	338 (65)
COPD	0 (0)	145 (2.9)	161 (6.3)	122 (10.6)	110 (21.15)

CHD, Coronary heart diseases; COPD, Chronic obstructive pulmonary diseases.

 The baseline characteristics of the participants stratified by MM are demonstrated in Table 4. The frequency of MM was significantly higher in female, rural, unmarried, and illiterate participants. Compared with the subjects in the 3^rd^ METs tertile and the 5^th^ WSI quintile, the prevalence of MM was significantly higher in subjects with low physical activity (1^st^ METs tertile) and low socioeconomic status (1^st^ WSI quintile).

**Table 4:** Baseline Characteristics of Participants Stratified by Multimorbidity

	**0 (n=5865)**	**1 (n=4923)**	**2 (n=2547)**	**3 (n=1151)**	**≥4 (n=520)**	***P***
	**No. (%)**	**No. (%)**	**No. (%)**	**No. (%)**	**No. (%)**	
Gender						< 0.001*
Male	3441 (51.3)	2069 (30.8)	779 (11.6)	327 (4.9)	96 (1.4)	
Female	2424 (29.2)	2854 (34.4)	1768 (21.3)	824 (9.9)	424 (5.1)	
Residential regions						0.05*
Urban residents	4149 (39.8)	3407 (32.7)	1754 (16.8)	771 (7.4)	350 (3.4)	
Rural residents	1717 (37.5)	1517 (33.2)	791 (17.3)	380 (8.3)	170 (3.7)	
Marital status						< 0.001*
Not married	336 (30.6)	323 (29.4)	214 (19.5)	144 (13.1)	81 (7.4)	
Married	5529 (39.8)	4600 (33.1)	2333 (16.8)	1007 (7.2)	439 (3.2)	
Education level						< 0.0001**
Illiterate	632 (25.2)	756 (30.1)	573 (22.9)	358 (14.3)	188 (7.5)	
Primary school	2150 (36.7)	2002 (34.1)	1054 (18)	440 (7.5)	213 (3.6)	
Diploma	2360 (44.4)	1777 (33.5)	769 (14.5)	297 (5.6)	111 (2.1)	
University	723 (54.6)	388 (29.3)	151 (11.4)	53 (4)	8 (0.6)	
Physical activity level (METs)						< 0.001**
Low	1599 (31.8)	1606 (31.9)	990 (19.7)	546 (10.9)	291 (5.8)	
Moderate	1785 (35.8)	1717 (34.5)	918 (18.4)	403 (8.1)	158 (3.2)	
High	2481 (49.7)	1601 (32.1)	637 (12.8)	202 (4)	71 (1.4)	
Quintiles of wealth index						< 0.0001**
1 (poorest)	1271 (36.5)	1151 (33.1)	602 (17.3)	319 (9.2)	137 (3.9)	
2	863 (34.1)	809 (32)	469 (18.5)	254 (10)	136 (5.4)	
3	1168 (38.2)	980 (32.1)	559 (18.3)	238 (7.8)	111 (3.6)	
4	1335 (42.7)	1087 (34.8)	480 (15.4)	159 (5.1)	64 (2)	
5 (richest)	1228 (43.6)	896 (31.8)	437 (15.5)	181 (6.4)	72 (2.6)	
Current smoking status						< 0.001**
No smoker	4080 (35.8)	3830 (33.6)	2096 (18.4)	948 (8.3)	447 (3.9)	
Ex-Smoker	497 (39.9)	417 (33.5)	187 (15)	109 (8.7)	36 (2.9)	
Smoker	1166 (56.1)	577 (27.8)	224 (10.8)	80 (3.8)	32 (1.5)	
Smoker other tobacco products (water pipe, hookah, pipe, etc)	122 (43.6)	99 (35.4)	40 (14.3)	14 (5)	5 (1.8)	
Sleep duration (hour/day)						0.15**
≤ 6.5	1756 (37.2)	1556 (33)	846 (17.9)	379 (8)	180 (3.8)	
6.6–7.3	1257 (43.5)	949 (32.8)	433 (15)	172 (6)	79 (2.7)	
7.4–8	1730 (41.6)	1360 (32.7)	669 (16.1)	279 (6.7)	119 (2.9)	
≥ 8	1122 (34.6)	1058 (32.7)	597 (18.4)	321 (9.9)	142 (4.4)	
Age (y), mean ± SD	47.41 ± 8.90	48.94 ± 9.05	52.36 ± 8.98	55.03 ± 8.50	56.25 ± 7.54	< 0.001***

METs, metabolic equivalent of task. * Chi-square test; ** Kruskal-Wallis H; *** One way ANOVA.

Table 5 presents the ordinal logistic regression analyses of the factors related to MM in the Azar Cohort Study population. The subjects in the 56–70 years age group showed significantly higher MM risk (OR = 3.44; 95% CI: 3.16–3.75) compared to those in the 35–45 years age group.

**Table 5:** Ordinal Logistic Regression Analysis of Factors Associated with Multimorbidity in the Azar Cohort Population

	**Unadjusted OR (95% CI)**	***P*** ** Value**	**Adjusted OR**^a^ ** (95% CI)**	***P *** **Value**
Age (y)				
35–45	Reference			
46–55	1.83 (1.71–1.96)	< 0.001	1.85 (1.72–1.99)	< 0.001
56–70	3.31 (3.07–3.57)	< 0.001	3.44 (3.16–3.75)	< 0.001
Gender				
Male	Reference			
Female	2.58 (2.43–2.74)	< 0.001	2.77 (2.59–2.96)	< 0.001
Residential regions				
Rural residents	1.09 (1.03–1.17)	0.003	0.96 (0.90–1.03)	0.31
Urban residents	Reference			
Marital status				
Not married	1.73 (1.54–1.94)	< 0.001	1.00 (0.88–1.12)	0.99
Married	Reference			
Education level				
Illiterate	4.07 (3.58–4.62)	< 0.001	1.74 (1.50–2.02)	< 0.001
Primary school	2.10 (1.88–2.36)	< 0.001	1.62 (1.43–1.83)	< 0.001
Diploma	1.50 (1.33–1.68)	< 0.001	1.39 (1.23–1.57)	< 0.001
University	Reference		-	
Quintiles of wealth index				
1 (poorest)	1.36 (1.24–1.49)	< 0.001	1.17 (1.07–1.29)^b^	< 0.001
2	1.57 (1.42–1.74)	< 0.001	1.34 (1.21–1.48)^b^	< 0.001
3	1.27 (1.16–1.40)	< 0.001	1.20 (1.09–1.32)^b^	< 0.001
4	0.97 (0.89–1.07)	0.61	1.05 (0.96–1.16)^b^	0.26
5 (richest)	Reference			
Physical activity level (METs)				
Low	2.35 (2.19–2.53)	< 0.001	1.89 (1.75–2.05)	< 0.001
Moderate	1.80 (1.67–1.93)	< 0.001	1.33 (1.23–1.44)	< 0.001
High	Reference			
Sleep duration				
≤ 6.5	0.87 (080–0.94)	0.001	1.02 (0.93–1.10)	0.67
6.6–7.3	0.65 (0.60–0.72)	< 0.001	0.82 (0.74–0.90)	< 0.001
7.4–8	0.72 (0.66–0.78)	< 0.001	0.85 (0.78–0.92)	< 0.001
> 8	Reference			
Current smoking status				
No smoker	Reference			
Ex-smoker	0.90 (0.84–0.96)	0.002	1.23 (1.14–1.32)	< 0.001
Smoker	0.60 (0.57–0.64)	< 0.001	0.94 (0.88–1.00)	0.07
Smoker other tobacco products (water pipe, hookah, pipe, etc)	0.78 (0.68–0.89)	0.001	1.25 (1.09–1.43)	0.001

METs, metabolic equivalent of task. ^a^ Adjusted for age, gender, education levels, marital status and wealth score index (WSI) if applicable. ^b^ Adjusted for age.

 According to the statistical method used, the MM risk increased significantly among illiterate individuals (OR = 1.74; 95% CI: 1.50–2.02), and females (OR = 2.77; 95% CI 2.59–2.96). Also, Table 5 shows that the subjects found within the lowest tertile of physical activity level (OR = 1.89; 95% CI: 1.75–2.05) showed higher MM risk than those in the highest level. Findings regarding current smoking status indicated that being an ex-smoker or smoker of other types of tobacco significantly increased the risk of MM in the research population. Finally, MM risk in subjects sleeping 6.6–7.3 and 7.4–8 hours a day decreased by 0.82 [0.74–0.90] and 0.85 [0.78–0.92], respectively, compared to those sleeping more than 8 hours a day.

## Discussion

 The findings of this cross-sectional study indicated that 28.1% of the total population had MM ( ≥ 2 CDs). The prevalence of MM varies across a wide range in different studies. In the Golestan Cohort Study conducted by Ahmadi et al, MM occurred in 19.4% of individuals aged 40–70 years.^16^ In another study, according to the data provided by health insurance organizations, MM was reported in 21.1% of the included population.^17^ In the elderly Kurdish population ( > 50 years), MM was seen in 36.1% of participants.^18^ Moreover, Aoki et al studied the pattern of MM in the Japanese population, noting the prevalence of MM to be 29.9% among all participants and 62.8% among the elderly ( ≥ 65 years).^19^ Additionally, in a cohort study in Germany, the prevalence of MM was 67.3% in the 50–70 years age group.^20^ The difference observed in the prevalence of MM in studies may be due to differences in sample size, the types of CDs considered as MM components, methodology, and ethnicity.

 In this study, the most common CDs were obesity, hypertension, depression, and diabetes. Moreover, the most co-occurring CDs were obesity, diabetes, and depression. Our findings are in line with previous studies that reported hypertension, diabetes, dyslipidemia, and obesity as the most common CDs in the population.^21^ Also, the most prevalent CDs reported by Blümel et al were hypertension, arthrosis, diabetes, and depression.^22^ In this regard, Read et al found that the risk of depression was twice in patients with MM compared with subjects without MM.^23^

 As indicated in previous studies, there is a mutual association between depression and CDs.^24^ However, the mechanism of this bidirectional relationship is not adequately recognized. Some studies express that the complication of CDs, including disability, decreased quality of life,^25^ pain,^26^ and beliefs about the disease and the adapting manners are involved in increasing the risk of depression.^27^ On the other hand, people with depression are less likely to follow treatment protocols for their CD, which increases their risk of developing MM and leaves them with poor control over their illness.^28^ Nevertheless, disorders in both metabolic and immune-inflammatory pathways, which occur in many CDs, are associated with depression.^29^ Therefore, depression should be considered an important associated factor in patients with CDs.

 In our study, the risk of MM in older subjects and females was higher than in the younger subjects and males. In studies similar to ours, age and gender were positively associated with MM.^16,17^ In this regard, Boutayeb et al reported that the female gender and advanced age are predictor factors of MM in WHO Eastern Mediterranean countries.^30^ However, in some studies, there were no discrepancies between the genders in the prevalence of MM.^21,31^ For instance, the EpiChron Cohort Study indicated that the number of CDs increased with age in both genders.^21^

 Our analysis revealed education level to be inversely associated with MM. Similarly, Blümel et al observed an increase of 40% in the risk of MM among low-educated women who had an unqualified job.^22^ Moreover, Johnson-Lawrence et al showed that the risk of MM in those aged 60–64 years with a bachelor’s degree (or higher) was lower relative to less-educated people.^32^ Since education is a key factor in social elements such as employment, health insurance, and housing, lower education levels may lead to decreased income, poor living conditions, and psychological stress. These conditions can prevent people from exercising proper health practices such as a healthy diet, physical exercise, and access to preventive healthcare, resulting in a higher risk of MM.^33^

 In previous studies, socioeconomic status was correlated with MM. Specifically, the prevalence of MM was higher in low socioeconomic groups.^17,34^ In our study, the frequency of MM decreased with increasing WSI, confirming the findings of the referenced studies. It has been suggested that the higher prevalence of MM in the population with low WSI can be attributed to less knowledge about symptoms of CDs, fewer checkups, and the consumption of unhealthy foods.^35,36^

 Our study also indicated that ex-smokers and smokers of other tobacco products had a higher risk of MM. Since we had asked the participants about their current smoking status, cessation of smoking after developing CDs may explain this finding.^37^ In previous studies, a smoking history has been documented as a risk factor for MM.^16,38^ Similarly, long-term water pipe and hookah users have greater odds of developing CDs such as hypertension, hyperlipidemia, hyperglycemia, obesity, abdominal obesity, and cancer.^39-41^

 In this study, we found that the risk of MM was greater among inactive participants (1^st^ tertile METs) than in the active population. Moreover, the odds of MM significantly decreased in participants who sleep for 6.6–8 hours a day compared with those who sleep more than 8 hours per day. In other words, physical activity and sleeping habits had a significant effect on the prevalence of MM. Similar to the present study, Ruiz-Castel et al noted that the chance of having two, or three or more chronic conditions increased by 7.30 and 6.79 times, respectively, in participants who sleep < 6 hours/day.^42^ In another study, Nicholson et al reported that the risk of MM was higher in participants who sleep either < 6 hours/day or > 8 hours/day.^43^ According to the literature, a wide range of adverse outcomes are associated with both limited and extensive sleep durations.^44,45^ Among them are overall mortality, diabetes mellitus, adverse cardiovascular events, and poor disease consequences.^46^ It appears that the association between sleeping habits and the number of CDs is reciprocal. To elaborate, the sleeping habits in subjects with CDs are subject to change by the pain caused by some CDs, medications/treatments, and mood disorders.^47^ On the other hand, it is well known that sustained sleep deprivation induces adverse effects on the cardio-metabolic, endocrine, and immune systems, and inflammatory pathways.^48,49^ Moreover, shorter sleep durations could modify the circadian rhythm and alter hormonal systems (e.g., insulin resistance and decreased leptin).^50^

 The association between physical activity and the prevalence of MM in the present study agrees with the findings of Christofoletti et al, where the frequency of co-existing CDs was greater among those who had more leisure time and watched TV ≥ 2 hours/day.^20^ Moreover, Ryan et al noted a significant association between physical activity and MM.^51^ However, other studies found no significant association between physical activity and MM.^52,53^ In explaining this discrepancy, low physical activity may be a consequence of MM rather than its risk factor or determinant. In this regard, it has been reported that people with MM are more likely to experience reduced physical activity, resulting in a cycle of poor health. Function is lost due to this detrimental cycle, leading to diminished ability to take up physical activity and exercise.^52^

 The main strength of this study is the use of data from a large, population-based cohort study. Similar to all studies, this study had its limitations, including the type of study (i.e., cross-sectional) and the illnesses reported by each individual, which is likely to bias the data due to differences in the level of literacy and information retrieval from the participants.

 In conclusion, our study is valuable since it examined the MM patterns in terms of multiple economic, social, and epidemiologic aspects in parts of Iran where the general population is at an increased risk of depression and metabolic syndrome components (obesity, hypertension, and diabetes). This increased risk is specifically prominent among the elderly, females, ex-smokers, waterpipe/pipe smokers, and those with low socioeconomic status, low education level, improper sleeping habits, or inactivity. Finally, it should be noted that the reduction in MM is possible by promoting public health from an early age among people of a wide range of socioeconomic conditions, provided that the necessary health support is offered to the aging Iranian population.
